# Supplementary material for: Genomic Analysis and Assessment of Melanin Synthesis in Amorphotheca resinae KUC3009
Source: J Fungi (Basel). 2021 Apr 12;7(4):289. doi: 10.3390/jof7040289 (PMC8069745; doi:10.3390/jof7040289)
Supplement: Supplementary file 1 [file jof-07-00289-s001.pdf]

Supplementary material

Genomic analysis and assessment of melanin synthesis in *Amorphotheca resinae* KUC3009

Jeong-Joo Oh [1], Young Jun Kim [2], Jee Young Kim [1], Sun Lul Kwon [1], Changsu Lee [3], Myeong-Eun Lee [4], Jung Woo Kim [5], and Gyu-Hyeok Kim [1,*]

[1] Division of Environmental Science & Ecological Engineering, College of Life Sciences & Biotechnology, Korea University, 145, Anam-ro, Seongbuk-gu, Seoul 02841, Republic of Korea.

[2] Life Science and Biotechnology Department, Underwood Division, Underwood International College, Yonsei University, Seoul, 03722, Republic of Korea.

[3] Microbiology and Functionality Research Group, World Institute of Kimchi, Gwangju 61755, Republic of Korea

[4] Department of Biotechnology, College of Life Sciences & Biotechnology, Korea University, 145, Anam-ro, Seongbuk-gu, Seoul 02841, Republic of Korea.

[5] Department of Biomedical Engineering, Sungkyunkwan University, Suwon, 2066 Seobu-ro, Jangan-gu, Suwon 16419, Republic of Korea.

*** Corresponding author**

Tel.: +82-2-3290-3014

Fax: +82-2-3290-9753

E-mail: lovewood@korea.ac.kr

Table S1. Assessment of genome completeness using BUSCO software.

Classification	Number of BUSCO groups
Complete	1300
Complete and single-copy BUSCOs	1294
Complete and duplicated BUSCOs	6
Fragmented BUSCOs	5
Missing BUSCOs	10
**Total BUSCOs groups searched**	**1315**

Table S2. Comparisons of genome features between *A. resinae* KUC3009 and the close relatives in the family *Myxotrichaeae*.

**Genome features**	***A. resinae*** **ATCC 22711** **(Elena et al, 2017)**	***O. maius*** **Zn** **(Kohler et al, 2015)**
Number of contigs	309	433
Length of the largest contigs	775,397	1,918,210
Average length of contigs	92,103.7	106,799.1
Total length of contigs	28,460,037	46,243,990
N50	216,801	454,872
Genome coverage	42.9×	28.7×
G+C content (%)	47.6	47.1
Average nucleotide identity (%) with *A. resinae* KUC3009	98.24	72.83

Table S3. Putative tyrosinase genes found in the *A. resinae* genome.

Gene name	Contig	Protein ID	Partial or Complete	Predicted signal peptide
Putative tyrosinase gene 1	2	LOCUS_001266-RA	Partial	-
Putative tyrosinase gene 2	3	LOCUS_002768-RA	Complete	None
Putative tyrosinase gene 3	7	LOCUS_006114-RA	Partial	-
Putative tyrosinase gene 4	13	LOCUS_009186-RA	Partial	-

* - indicates no search was conducted due to partial properties of gene.

Table S4. Putative multi-copper oxidase genes found in the *A. resinae* genome.

Gene name	Contig	Protein ID	Partial or Complete	Predicted signal peptide
Putative MCO 1	2	LOCUS_001545-RA	Complete	None
Putative MCO 2	2	LOCUS_001550-RA	Complete	Sec/SPI (*p*: 0.9514)
Putative MCO 3	2	LOCUS_001570-RA	Partial	-
Putative MCO 4	2	LOCUS_001570-RB	Partial	-
Putative MCO 5	2	LOCUS_001598-RA	Partial	-
Putative MCO 6	2	LOCUS_001762-RA	Partial	-
Putative MCO 7	3	LOCUS_002688-RA	Complete	Sec/SPI (*p*: 0.9982)
Putative MCO 8	4	LOCUS_003173-RA	Complete	Sec/SPI (*p*: 0.9818)
Putative MCO 9	4	LOCUS_003552-RA	Complete	Sec/SPI (*p*: 0.9761)
Putative MCO 10	6	LOCUS_005496-RA	Partial	-
Putative MCO 11	7	LOCUS_005953-RA	Partial	-
Putative MCO 12	7	LOCUS_006220-RA	Partial	-
Putative MCO 13	7	LOCUS_006246-RA	Partial	-
Putative MCO 14	8	LOCUS_006345-RA	Complete	None
Putative MCO 15	8	LOCUS_006346-RA	Partial	-
Putative MCO 16	12	LOCUS_008739-RA	Complete	None

* - indicates no search was conducted due to partial properties of gene.
